# Prevalence and predictors of sex under the influence of psychoactive substances among young people in informal settlements in Kampala, Uganda

**DOI:** 10.1186/s12889-023-15679-8

**Published:** 2023-05-02

**Authors:** Tonny Ssekamatte, Aisha Nalugya, Richard K. Mugambe, Brenda Wagaba, Doreen Nakalembe, Aloysius Mutebi, Richard Asaba Bagonza, Arnold Tigaiza, Winnifred K. Kansiime, Richard Ssempala, Solomon T. Wafula, John Bosco Isunju, Esther Buregyeya

**Affiliations:** 1 Department of Disease Control and Environmental Health, School of Public Health, College of Health Sciences, Makerere University , P.o Box 7072, Kampala, Uganda; 2 Department of Health Policy Planning and Management, School of Public Health, College of Health Sciences, Makerere University, P.o Box 7072, Kampala, Uganda; 3 School of Women & Gender Studies, College of Humanities and Social Sciences, Makerere University, P.o Box 7062, Kampala, Uganda; 4 Department of Economic Theory and Analysis, School of Economics, College of Business and Management Sciences, Makerere University, P.o Box 7062, Kampala, Uganda

**Keywords:** Risky sexual behaviour, Psychoactive substance use, Young people, Informal settlements, Kampala Uganda

## Abstract

**Background:**

The use of psychoactive substances such as alcohol, heroin and marijuana is associated with negative health outcomes such as sexual violence and unintended pregnancies, and risky sexual behaviours. Although there is evidence linking psychoactive substance use and risky sexual behaviours such as inconsistent condom use and multiple sexual relationships, there is limited data on sex under the influence of psychoactive substances among young people. This study aimed to investigate the prevalence and predictors of sex under the influence of psychoactive substances among young people in informal settlements in Kampala, Uganda.

**Methods:**

A cross-sectional study was conducted among 744 sexually active young psychoactive substance users in informal settlements in Kampala, Uganda. Data were collected through face-to-face interviews using a digitalized structured questionnaire, preloaded on the Kobocollect mobile application. The questionnaire captured data on the socio-demographic characteristics of the respondents, history of psychoactive substance use, and sexual behaviours. Data were analysed using STATA Version 14.0. A modified Poisson regression model was used to determine the predictors of sex under the influence of psychoactive substances.. Adjusted prevalence ratios at a p-value value ≤ 0.05 with a 95% confidence interval were considered.

**Results:**

About 61.0% (454/744) of the respondents had had sex under the influence of psychoactive substances in the last 30 days. The predictors of sex under the influence of psychoactive substances were being female (PR 1.18, 95% CI: 1.04–1.34), being 20–24 years of age (PR: 1.22, 95% CI: 1.04–1.44), being married (PR 1.15, 95% CI: 1.01–1.31) or divorced/separated (PR 1.43, 95% CI: 1.26–1.61), not living with biological parents or guardians (PR 1.22, 95% CI: 0.99–1.50), earning 71 USD and below (PR 0.86, 95% CI: 0.79–1.03) and using alcohol (PR 1.43, 95% CI: 1.25–1.69), marijuana (PR 1.16, 95% CI: 1.02–1.31) and khat (PR 1.25, 95% CI: 1.10–1.42) in the last 30 days.

**Conclusion:**

The study found that a high proportion of sexually active young people in informal settlements in Kampala, Uganda had engaged in sex under the influence of psychoactive substances in the past 30 days. The study also identified several factors associated with sex under the influence of psychoactive substances, including being female, being aged 20–24 years, being married or divorced or separated, not living with biological parents or guardians, and using alcohol, marijuana, or khat in the past 30 days. Our findings suggest the need for targeted sexual and reproductive health programs that incorporate risk-reduction interventions aimed at reducing sex under the influence of psychoactive substances, especially among females and those who do not live with their parents.

## Background

The use of psychoactive substances remains a significant global public health challenge, especially among young people [1, 2], who are defined by the [3]World Health Organisation (WHO) as individuals aged 10–24 years [3]. Available evidence suggests that the use of psychoactive substances is likely to increase by 11% globally, and by 40% in Africa by 2030 [4]. The percentage increase in psychoactive substance use is likely to be much higher among young people especially in Africa [4], due to urbanisation and the fact that they currently use more drugs and at higher levels compared to adults and past generations [5]. This is amid a reduction in the proportion of young people who perceive psychoactive substances such as cannabis as harmful [4]. A recent systematic review indicated a prevalence of psychoactive substance use among young people in Sub-Saharan Africa as high as 41.6% [6]. Psychoactive substance use among young people remains on the increase in Uganda [7, 8]. A recent survey among adolescent boys and young men in Kampala indicated a 10.5% (6.6% among in-school and 21.9% among out-of-school adolescent boys and young men) prevalence of alcohol use in the past 30 days [9]. Abbo, Okello [10] also reported an 8.0% prevalence of current use of marijuana *(Cannabis sativa)*, 9.7% for khat *(Catha edulis)*, 9.9% for oral tobacco, and 9.6% for aviation fuel among students in secondary schools in Kampala.

Although data are limited, there is evidence of a much higher prevalence of psychoactive substance use in informal settlements [11], which in the current study, we define as urban residences characterised by poor housing and overcrowding, and high poverty levels [12]. The high prevalence of psychoactive substances in informal settlements is often driven by their widespread availability, peer pressure, curiosity, and psychological distress [13–15]. The use of psychoactive substances has been further aggravated by the COVID-19 pandemic [16]. The COVID-19 pandemic is associated with an increase in anxiety and depression due to a lack of social support, and loss of jobs and income among the residents of informal settlements. Data obtained from young people in Kampala also revealed that COVID-19 prevention measures such as lockdowns increased mental health challenges and limited the ability to meet basic needs [17]. The effects of COVID-19 culminated in an increase in the use of psychoactive substances as a coping mechanism [16, 18]. Despite the perceived benefits, the use of psychoactive substances is associated with a multitude of health risks, which have negative effects on the sexual and reproductive health of the users [19, 20]. Psychoactive substance use increases the probability of early sexual debut, trading sex for drugs or money, sexual violence, inconsistent condom use, multiple sexual partnerships, and sexual intercourse while intoxicated [21–24].

An increase in sexual expectancies such as pleasure and performance, courage to approach a sexual partner, and a reduction in sexual inhibitions such as a lack of confidence often drive the use of psychoactive substances among young people in informal settlements [12]. However, these substances impair cognition and decision-making leading to risky sexual behaviours such as sexual intercourse while intoxicated [25]. Sex under the influence of psychoactive substances compromises one’s ability to use condoms [11], and exacerbates the risk of sexual coercion and non-spousal sex [23, 26]. Consequently, this exposes psychoactive substance users to sexually transmitted infections (STIs) including HIV [7, 27, 28], and unintended pregnancies [29–31].

Despite evidence of the risks associated with sex under the influence of psychoactive substances, there are limited data on its prevalence and predictors, especially in low-and middle-income countries. Available data focus on other high-risk sexual behaviours such as inconsistent condom use, multiple sexual partnerships, transactional sex, and early sex debut [32–34]. Nonetheless, the few studies conducted in high-income countries indicate that adolescents who had their most recent experience of intercourse away from home and those who had problems enjoying sex were more likely than others to have sex while being intoxicated. Understanding the predictors of sex under the influence of psychoactive substances can inform sexual and reproductive health interventions targeting young users of psychoactive substances. The current study used theories of alcohol myopia [35] and expectancy [36] to understand the predictors of sex under the influence of psychoactive substances among young people. On the one hand, the alcohol myopia theory proposes that psychoactive substances limit an individual’s ability to consider the future consequences of their actions, and to regulate their reactive impulses, confining them to the present moment, without considering possible consequences [35]. On the other hand, expectancy theory focuses on the importance of internalized cultural and social expectations regarding the effects of psychoactive substances on sexual behaviour. In this theory, individuals’ expectations that substance use will offer better sexual experiences moderates their relationship to sexual behaviour, making sexual intercourse under the influence more likely and riskier as such expectations increase [36–39].

## Materials and methods

### Study design, setting, and population

This cross-sectional study used quantitative data collection methods to establish the prevalence and predictors of sex under the influence of psychoactive substances among young people in informal settlements in Kampala. Kampala is the largest urban centre and capital city in Uganda. It is the country’s economic hub and accounts for 80% of its industrial and commercial activities, and generates 65% of the national GDP [40]. Kampala has a total population of 1,507,080, 53% of which are females. The city is projected to have a total population of 1,927,400 by 2030 [40]. Kampala has a total of 57 informal settlements distributed among the five administrative divisions namely Central, Kawempe, Makindye, Lubaga and Nakawa. Kampala covers a total area of 189 km^2^ with 169 km^2^ of land and 19 km^2^ of water [40]. This study was conducted in 12 purposively selected informal settlements in 4 of Kampala’s divisions (Kawempe, Makindye, Lubaga, and Nakawa). The informal settlements included Kinawataka; Luzira; Luzira-Kirombe; Kitintale, Nalukolongo; Wankulukuku-Kabowa; Kamwokya; Bwaise; Wandegeya; Katwe-Kinyoro; Namuwongo-Soweto and Kyebando.The geographical representation of the entire city was considered while selecting each of these informal settlements. Kampala’s informal settlements are characterised by high levels of psychoactive substance use, poverty and unemployment [41–43]. 

### Eligibility criteria

To be eligible to participate in the survey, the prospective respondent had to be a current psychoactive substance user aged 18–24 years and residing in one of the informal settlements of Kampala. To be considered a current user, the prospective respondent must have consumed or used alcohol, marijuana, khat, oral tobacco, and/or heroin, which are the most common psychoactive substances in the study area [44–46]. Although the age group of 12–17 years is the critical period for the initiation of psychoactive substance use, we recruited young people aged 18–24 years, because peak levels of drug and substance use are observed during this age group [4]. Individuals aged 18–24 years also have more autonomy in engaging in psychoactive substance use and sexual activity than those aged below 18 years, since they are considered minors. Recent evidence from Kampala also reveals that psychoactive substance use among young people in Kampala is highest during late adolescence (18–19 years) and young adulthood (20–24 years) [9]. Although ucommon, respondents who were intoxicated were rescheduled for the interview to a day they were sober. Interviews were mainly conducted during the morning hours because it was the period when the majority of the respondents were not under the influence of psychoactive substances.

### Sample size and sampling procedure

Data presented in the current manuscript are part of the study titled “High-risk sexual behaviours of young psychoactive substance users in Kampala’s informal settlements, Uganda” which was conducted between June and July 2019 [12, 47–49]. The sample size was calculated using the Kish Leslie formula for cross-sectional studies [50]. We considered a conservative prevalence of high-risk sexual behaviours among young psychoactive-substance users of 50.0% given the limited evidence on this subject among the study population, a margin of error of 5% corresponding to a 95% level of confidence, and a design effect of 2.0. A total of 768 participants was obtained. The analysis in the current manuscript, however, was restricted to the 744 young psychoactive substance users who were sexually active. A respondent was considered sexually active if they had had sexual intercourse at least once in the last 30 days [51]. We chose this period to reduce chance of recall bias.

Details of the sampling methodology have been reported in our earlier publications [12, 47–49]. Briefly, study participants were selected using the respondent-driven sampling (RDS) technique. RDS is a sampling strategy involving chain-referral recruitment driven by peers [52, 53]. This method is recommended for hard-to-reach populations such as users of psychoactive substances in informal settlements [52, 53]. First, we contacted individuals from an earlier study [54] who supported the initial recruitment of study participants. These individuals enrolled four young psychoactive substance users (2 males and 2 females) in each informal settlement, for a total of 48 seeds. The recruitment process was guided by peer educators, local leaders, and staff of non-governmental organisations working on psychoactive substance programs to ensure that the selected seeds were young psychoactive substance users. Research Assistants (RAs) (who were trained on signs of intoxication) screened each respondent before the interview to ensure that they were not under the influence of psychoactive substances. The intention was to ensure that the respondents were sufficiently sober to provide informed consent prior to participation in the study. Intoxication can impair cognition, judgement, and the capacity to understand research and make decisions regarding participation. Henceforth, we identified impairments thought to result from intoxication, such as the physical manifestations of intoxication (staggering gait, slurred speech, glazed eyes), as reported in previous studies [55]. Thereafter, the RAs administered a digitalized structured questionnaire to the eligible users of psychoactive substances. The respondents received 3 coupons to distribute and recruit 3 of their peers who were eligible for the study. Primary seeds made the first wave whereas their recruits produced the second wave. The coupons clearly stated information about the study including the study aim, location, contact details of the principal investigator, hours of operation, coupon identification numbers, and the start and expiry dates. The expiry dates enabled us to determine how many valid and expired coupons were still in circulation, although, participants presenting expired coupons were still enrolled if they met the eligibility criteria.

### Variables measurement

Our outcome variable was sex under the influence of psychoactive substances in 30 days prior to the interview. Sex under the influence of psychoactive substances was defined as sexual intercourse when a partner is intoxicated [56]. Responses were categorized as Yes (1) or No (0). Respondents were asked if they had engaged in sex under the influence of psychoactive substances in the last 30 days. It was coded as 1 if a young psychoactive substance user responded with “Yes” and 0 if they responded with “No”. The independent variables included socio-demographic characteristics such as age, marital status, living arrangement, average monthly income, highest level of formal education, duration of stay in informal settlements, and history of psychoactive substance use. To obtain the average monthly income, respondents were asked how much they earned on average. The data on monthly income and age were continuous. Age was caterigozed as 18–19 and 20–24 years. The use of psychoactive substances was measured over a recall period of 12 months and 30 days. First, respondents were asked if they had used any psychoactive substance in the last 12 months, and later on, asked if they had used a psychoactive substance in the past 30 days. The psychoactive substances of interest in this study included alcohol, marijuana, oral tobacco, khat, and heroin. These substances were chosen due to their wide spread availabilty in informal settlements in Kampala, Uganda.

### Quality control, data collection procedures and tools

Experienced RAs with a minimum of a bachelor’s degree in social or health sciences were recruited to support the data collection exercise. RAs were recruited based on their ability to read and write Luganda (local language) and English, experience working in informal settlements, and conducting community-based sexual and reproductive health surveys. They were oriented on the study protocol, including the study objectives and methodology. They were also trained on the questionnaire/tool, interviewing skills, and ethical considerations. To enhance quality data collection, the questionnaire was translated to Luganda and pretested in an informal settlement in Kajjansi, Wakiso district. The goal of the pretesting activity was to ensure that the questions were clearly understood by the respondents and to enable the RAs familiarize with the data collection tool. During field work, RAs were supervised by the lead investigator (T.S) to ensure that they followed the study protocol and ethical procedures while interviewing the respondents. During data collection, a digitalized structured questionnaire was used to obtain detailed information on socio demographics, history of psychoactive substance use and the respondents’ sexual behaviour while under the influence of substances. The KoboToolbox server was used to design the questionnaire, which was later uploaded on to the mobile application, pre-installed on android-enabled smart devices. The Kobo Collect data entry screen was designed with skips and restrictions to eliminate missingness of data and inconsistencies. While in the field, RAs were required to upload data to the cloud server on a daily basis for quality control purposes.

### Data management and analysis plan

Data were dowloaded from the server and cleaned using a combination of microsoft Excel and STATA version 14.0. Data were cleaned by detecting and correcting (or removing) inacurracies and errors, removing duplicates and harmonization of data. Descriptive statistics (frequencies, means, medians, standard deviation and interquartile range) were done to summarize the data. Results were presented in tables and figures as appropriate. Bivariable Poisson regression was done to determine the association between independent variables and sex under the influence of psychoactive substances. This is because the prevalence of sex under the influence of alcohol was greater than 10%, and using odds ratios would over-estimate the strength of association. All variables whose p-values were less than 0.2 were included in multivariate ‘modified’ Poisson regression model. A p ≤ 0.05 was considered statistically significant. Adjusted prevalence ratios at a 95% confidence interval were used to measure the strength of the association.

## Results

### Socio-demographic characteristics of the respondents

A sample of 744 young psychoactive substance users was analysed. More than three-quarters, 78.0% (580/744) of the respondents were males, 76.6% (570/744) were aged 20–24 years and 58.3% (434/744) (58.6% for those aged 18–19 years and 58.2% for those aged 20–24 years) had attained formal education above the primary level. Nearly half, 45% (335/744) (49.0% for males and 31.1% for females ) of the respondents had stayed in the informal settlements for more than 10 years. More than a tenth, 14.7% (109/744) of the respondents were living with their biological parents/guardians (Table 1). With regard to the main source of income; 39.1% (291/744) of the respondents engaged in petty trade, 20.3% (151/744) were casual labourers, 9.8% (73/744) had formal employment, 8.1% (60/744) were involved in transport business (e.g. taxi drivers or motorcycle riders), 5.0% (37/744) were bar attendants, 5.0% (37/744) were female sex workers, 2.4% (18/744) were psychoactive substance dealers and 1.6% (12/744) were farmers. Additionally, 7.1% (53/744) were unemployed while 1.6% (12/744) were students. Sex work was the main source of income for 22.6% (37/164) of the female respondents.

**Table 1:** Socio-demographic characteristics of young psychoactive substance users

Variable	Attribute	Overall (N= 744)	Gender		Age- category (Years)	
			Males (n = 580)	Females (n = 164)	18–19 (n = 174)	20–24 (n = 570)
Division	Kawempe	349 (46.9)	260 (44.8)	89 (54.3)	52 (29.9)	297 (52.1)
	Makindye	133 (17.9)	113 (19.5)	20 (12.2)	45 (25.9)	88 (15.4)
	Nakawa	157 (21.1)	124 (21.4)	33 (20.1)	50 (28.7)	107 (18.8)
	Rubaga	105 (14.1)	83 (14.3)	22 (13.4)	27 (15.5)	78 (13.7)
Sex	Male	580 (78.0)			140 (80.5)	440 (77.2)
	Female	164 (22.0)			34 (19.5)	130 (22.8)
Age category (years)	18–19	174 (23.4)	140 (24.1)	34 (20.7)		
	20–24	570 (76.6)	440 (75.9)	130 (79.3)		
Highest level of formal education	Primary and below	310 (41.7)	229 (39.5)	81 (49.4)	72 (41.4)	238 (41.8)
	Above Primary	434 (58.3)	351 (60.5)	83 (50.6)	102 (58.6)	332 (58.2)
Current marital status	Single	514 (69.1)	416 (71.7)	98 (59.8)	153 (87.9)	361 (63.3)
	Married	162 (21.8)	119 (20.5)	43 (26.2)	14 (8.1)	148 (26.0)
	Divorced/separated	68 (9.1)	45 (7.8)	23 (14.0)	7 (4.0)	61 (10.7)
Average monthly income (1USD = 3550 UGX)	0-250000	477 (64.1)	348 (60.0)	129 (78.7)	126 (72.4)	351 (61.5)
	250,001–500,000	204 (27.4)	176 (30.3)	28 (17.0)	39 (22.4)	165 (29.0)
	Above 500,000	63 (8.5)	56 (9.7)	7 (4.3)	9 (5.2)	54 (9.5)
Duration of staying in the area (In years)	0–5	264 (35.5)	175 (30.2)	89 (54.3)	73 (42.0)	191 (33.5)
	6–10	145 (19.5)	121 (20.8)	24 (14.6)	22 (12.6)	123 (21.6)
	More than 10	335 (45.0)	284 (49.0)	51 (31.1)	79 (45.4)	256 (44.9)
Living with biological parents or guardians	Yes	109 (14.7)	91 (15.7)	18 (11.0)	53 (30.5)	56 (9.8)
	No	635 (85.3)	489 (84.3)	146 (89.0)	121 (69.5)	514 (90.2)

### History of psychoactive substance use among the respondents

Overall, 84.5% (629/744) of the respondents had ever used alcohol. Majority, 81.2% (604/744) and nearly three quarters, 74.3% (553/744) had used alcohol in the last 12 months and 30 days respectively. Nearly two thirds, 62.2% (463/744) of the respondents had ever used marijuana and 57.5% (428/744) had used marijuana in the last 12 months. More than half, 51.9% (386/744) of the respondents had used marijuana in the last 30 days. Less than a tenth, 5.9% (44/744) of the respondents had ever used heroin. Only 1.8% (14/744) of the respondents had used heroin in the last 30 days (Table 2).

**Table 2:** History of psychoactive substance use among the respondents

Variable	Attribute	Overall (N = 744)	Gender		Age category (Years)	
			Males (n = 580)	Females (n = 164)	18–19 (n = 174)	20–24 (n = 570)
**Alcohol use**						
Ever used alcohol	Yes	629 (84.5)	474 (81.7)	155 (94.5)	130 (74.7)	499 (87.5)
	No	115 (15.5)	106 (18.3)	9 (5.5)	44 (25.3)	71 (12.5)
Used alcohol in the last 12 months	Yes	604 (81.2)	452 (77.9)	152 (92.7)	122 (70.1)	482 (84.6)
	No	140 (18.8)	128 (22.1)	12 (7.3)	52 (29.9)	88 (15.4)
Used alcohol in the last 30days	Yes	553 (74.3)	408 (70.3)	145 (88.4)	107 (61.5)	446 (78.3)
	No	191 (25.7)	172 (29.7)	19 (11.6)	67 (38.5)	124 (21.7)
**Marijuana use**						
Ever used Marijuana	Yes	463 (62.2)	389 (67.1)	74 (45.1)	111 (63.8)	352 (61.7)
	No	281 (37.8)	191 (32.9)	90 (54.9)	63 (36.2)	218 (38.3)
Used Marijuana in the last 12 months	Yes	428 (57.5)	360 (62.1)	68 (41.5)	101 (58.1)	327 (57.4)
	No	316 (42.5)	220 (37.9)	96 (58.5)	73 (41.9)	243 (42.6)
Used Marijuana in the last 30 days	Yes	386 (51.9)	326 (56.2)	60 (36.6)	87 (50.0)	299 (52.5)
	No	358 (48.1)	254 (43.8)	104 (63.4)	87 (50.0)	271 (47.5)
**Khat use**						
Ever used Khat	Yes	475 (63.8)	395 (68.1	80 (48.8)	124 (71.3)	351 (61.6)
	No	269 (36.2)	185 (31.9)	84 (51.2)	50 (28.7)	219 (38.4)
Used Khat in the last 12 months	Yes	450 (60.5)	374 (35.5)	76 (46.3)	119 (68.4)	331(58.1)
	No	294 (39.5)	206 (35.5)	88 (53.7)	55 (31.6)	239 (41.9)
Used Khat in the last 30 days	Yes	409 (55.0)	343 (59.1)	66 (40.2)	108 (62.1)	301 (52.8)
	No	335 (45.0)	237(40.9)	98 (59.8)	66 (37.9)	269 (47.2)
**Oral tobacco use**						
Ever used Oral tobacco	Yes	120 (16.1)	94 (16.2)	26 (15.9)	21 (12.1)	99 (17.4)
	No	624 (83.9)	486 (83.8)	138 (84.1)	153 (87.9)	471 (82.6)
Used oral tobacco in the last 12 months	Yes	91 (12.2)	67 (11.4)	24 (14.6)	16 (9.2)	75 (13.2)
	No	653 (87.8)	513 (88.6)	140 (85.4)	158 (90.8)	495 (86.8)
Used oral tobacco in the last 30 days	Yes	67 (9.0)	47 (8.1)	20 (12.2)	11 (6.3)	56 (9.8)
	No	677 (91.0)	533 (91.9)	144 (87.8)	163 (93.7)	514 (90.2)
**Heroin use**						
Ever used heroin	Yes	44 (5.9)	34 (5.9)	10 (6.1)	8 (4.6)	36 (6.3)
	No	700 (94.1)	546 (94.1)	154 (93.9)	166 (95.4)	534 (93.7)
Used heroin in the last 12 months	Yes	27 (3.6)	18 (3.1)	9 (5.5)	2 (1.2)	25 (4.4)
	No	717 (96.4)	562 (96.9)	155 (94.5)	172 (98.8)	545 (95.6)
Used heroin in the last 30 days	Yes	14 (1.8)	9 (1.5)	5 (3.0)	1 (0.6)	13 (2.3)
	No	730 (98.2)	571 (98.5)	159 (97.0)	173 (99.4)	557 (97.7)

### Sexual behaviours, and prevalence of sex under the influence of psychoactive substances stratified by socio-demographic characteristics

About 44.8% (344/744) of the respondents reported that either themselves or their partners had used a psychoactive substance during their last sexual intercourse. About 20.8% (46/164) of the females had had their last sexual intercourse with a sex client. Nearly a tenth, 8.6% (50/580) and 6.7% (39/580) of the males had had their last sexual intercourse with a female sex worker and casual acquittance respectively.

About 61.0% (454/744) of the respondents had sex under the influence of psychoactive substances in the last 30 days. At bivariate, sex of the respondent was significantly associated with sex under the influence of psychoactive substances. More than half, 58.1% (337/580) of the males in this study had sex under the influence of psychoactive substances in the last 30 days compared to 71.3% (117/164) among females (p = 0.002). The age of the respondent was associated with sex under the influence of psychoactive substances in the last 30 days. Nearly two thirds, 65.3% (372/570) of the respondents aged 20–24 years had sex under the influence of psychoactive substances in the last 30 days compared to 47.1% (82/174) among respondents aged 18–19 years (p < 0.001). The marital status of the respondent was also associated with sex under the influence of psychoactive substances in the last 30 days. About 59.1% (344/582) of the single respondents had sex under the influence of psychoactive substance in the last 30 days compared to 67.9% (110/162) among the married respondents (p < 0.001). Almost all, 97.4% (209/310) of the respondents who had attained primary education and below had sex under the influence of psychoactive substances in the last 30 days compared to 56.5% (245/434) of those who had attained education above the primary level (p = 0.002). Nearly two thirds, 63.8% (405/635) of the respondents who were not living with their biological parents or guardians had sex under the influence of psychoactive substances in the last 30 days compared to 45% (49/109) of the respondents who were still living with their biological parents or guardians (p < 0.001).There was no statistical difference between religion (p = 0.774) and average monthly income (p = 0.223), and sex under the influence of psychoactive substances in the last 30 days (Table 3).

**Table 3:** Prevalence of sex under the influence of psychoactive substances among young people in informal settlements in Kampala based on socio-demographic characteristics

Socio demographics	Attribute	Had sex under the influence of psychoactive substances drugs in the last 30 days		*χ*^2^ p-value
		Yes, n (%)	No, n (%)	
Sex	Male	337 (58.1)	243 (41.9)	
	Female	117 (71.3)	47 (28.7)	0.002
Age category (years)	18–19	82 (47.1)	92 (52.9)	
	20–24	372 (65.3)	198 (34.7)	p < 0.001
Highest level of education	Primary and below	209 (67.4)	101 (32.6)	
	Above primary	245 (56.5)	189 (43.5)	0.002
Current marital status	Divorced	59 (86.8)	9 (13.2)	
	Married	110 (67.9)	52 (32.1)	
	Single	285 (55.5)	229 (44.5)	p < 0.001
Religion	Catholic	180 (61.9)	111 (38.1)	
	Anglican	81 (64.3)	45 (35.7)	
	Muslim	128 (57.7)	94 (42.3)	
	Pentecostal	50 (62.5)	30 (37.5)	
	Other	15 (60.0)	10 (40.0)	0.774
Living with biological parents or guardians	Yes	49 (45.0)	60 (55.0)	
	No	405 (63.8)	230 (36.2)	p < 0.001
Average monthly income	UGX 0-250000	302 (63.3)	175 (36.7)	
(1USD = 3690 UGX)	UGX 250,001–500,000	117 (57.4)	87 (42.6)	
	Above 500,000	35 (55.6)	28 (44.4)	0.233
Duration of stay in informal settlement	0–5 years	151 (57.2)	113 (42.8)	
	6–10 years	86 (59.3)	59 (40.7)	
	More than 10 years	217 (64.8)	118 (35.2)	0.151

### Sex under the influence of psychoactive substances based on type of psychoactive substance

There was a significant association between using alcohol in the last 30 days and sex while under the influence of psychoactive substances. A significantly higher proportion, 67.3% (372/553) of respondents who had used alcohol in the last 30 days had sex under the influence of psychoactive substances compared to 32.7% (181/553), p < 0.001 who had not. There was a significant association between using marijuana in the last 30 days and sex while under the influence of psychoactive substances. A higher proportion, 66.3% (256/386) of respondents who had used marijuana in the last 30 days had sex under the influence of psychoactive substances compared to 33.3% (130/386), p = 0.002 who had not. There was no statistical difference between using heroin in the last 30 days and sex under the influence of psychoactive substances in the last 30 days (p = 0.420) (Table 4).

**Table 4:** Prevalence of sex under the influence of psychoactive substances among young people in informal settlements in Kampala based on history of use and type of psychoactive substance

History of substance use	Attribute	Had sex under the influence of psychoactive substances in the last 30 days		*χ*^2^ p-value
		Yes, n (%)	No, n (%)	
**Ever used a psychoactive substance**				
Alcohol	Yes	402 (63.9)	227 (36.1)	
	No	52 (45.2)	63 (54.8)	p < 0.001
Marijuana	Yes	299 (64.6)	164 (35.4)	
	No	155 (55.2)	126 (44.8)	0.011
Khat	Yes	312 (65.7)	163 (34.3)	
	No	142 (52.8)	127 (47.2)	0.001
Oral tobacco	Yes	87 (72.5)	33 (27.5)	
	No	367 (58.8)	257 (41.2)	0.005
Heroin	Yes	31 (70.5)	13 (29.5)	
	No	423 (60.4)	277 (39.6)	0.186
**Type of psychoactive substance used in the last 12 months**				
Alcohol	Yes	391 (64.7)	213 (35.3)	
	No	63 (45.0)	77 (55.0)	p < 0.001
Marijuana	Yes	279 (65.2)	149 (34.8)	
	No	175 (55.4)	141 (44.6)	0.007
Khat	Yes	296 (65.8)	154 (34.2)	
	No	158 (53.7)	136 (46.3)	0.001
Oral tobacco	Yes	69 (75.8)	22 (24.2)	
	No	385 (59.0)	268 (41.0)	0.002
Heroin	Yes	20 (74.1)	7 (25.9)	
	No	434 (60.5)	283 (39.5)	0.157
**Type of psychoactive substance used in the last 30 days**				
Alcohol	Yes	372 (67.3)	181 (32.7)	
	No	82 (42.9)	109 (57.1)	p < 0.001
Marijuana	Yes	256 (66.3)	130 (33.7)	
	No	198 (55.3)	160 (44.7)	0.002
Oral tobacco	Yes	51 (76.1)	16 (23.9)	
	No	403 (59.5)	274 (40.5)	0.008
Khat	Yes	277 (67.7)	132 (32.3)	
	No	177 (52.8)	158 (47.2)	p < 0.001
Heroin	Yes	10 (71.4)	4 (28.6)	
	No	444 (60.8)	286 (39.2)	0.420

### Predictors of sex under the influence of psychoactive substances

Female young psychoactive substance users had an 18% higher prevalence of sex under the influence of psychoactive substances in the last 30 days compared to their male counterparts (PR 1.18, 95% CI: 1.04–1.34). Young psychoactive substance users aged 20-24years had a 22% higher prevalence of sex under the influence of psychoactive substances in the last 30 days compared to those aged 18–19 years (PR: 1.22, 95% CI: 1.04–1.44). Married respondents had a 15% higher prevalence of sex under the influence of psychoactive substances in the last 30 days compared to their single counterparts (PR 1.15, 95% CI: 1.01–1.31). Respondents who earned an average monthly income between UGX 250,000-500,000 (PR 0.86, 95% CI: 0.79–1.03) had a 14% lower prevalence of sex under the influence of psychoactive substances in the last 30 days compared to those who earned an average monthly income of UGX 0-250,000. Alcohol use in the last 30 days was associated with a 43% higher prevalence of sex under the influence of psychoactive substances in the last 30 days (PR 1.43, 95% CI: 1.25–1.69). Marijuana use in the last 30 days was associated with a 16% higher prevalence of sex under the influence of psychoactive substances in the last 30 days (PR 1.16, 95% CI: 1.02–1.31). Khat use in the last 30 days was associated with a 25% higher prevalence of sex under influence of psychoactive substance in the last 30 days (PR 1.25, 95% CI: 1.10–1.42) (Table 5).

**Table 5:** Multi-variate analysis of predictors of sex under the influence of psychoactive substances among young people in informal settlements in Kampala, Uganda

Variable	Freq (N)	Had sex under the influence of drugs in the last 30 days		Unadjusted PRR (95% CI)	P value	Adjusted PRR (95% CI)	P value
		Yes n (%)	No n (%)				
**Sex **							
Male	580	337 (74.2)	243 (83.8)	1		1	
Female	164	117 (25.8)	47 (16.2)	1.22 (1.08–1.38)	0.001	1.18 (1.04–1.34)	**0.007**
**Age category (years)**							
18–19	174	82 (18.1)	92 (31.7)	1		1	
20–24	570	372 (81.9)	198 (68.3)	1.38 (1.17–1.63)	0.001	1.22 (1.04–1.44)	**0.014**
**Marital status**							
Single	514	285 (62.8)	229 (79.0)	1		1	
Married	162	110 (24.2)	52 (17.9)	1.22 (1.07–1.39)	0.001	1.15 (1.01–1.31)	**0.031**
Divorced/ separated	68	59 (13.0)	9 (3.1)	1.56 (1.38–1.76)	p < 0.001	1.43 (1.26–1.61)	**p < 0.001**
**Still living with parents/gurdians**							
Yes	109	49 (10.8)	60 (20.7)	1			
No	635	405 (89.2)	230 (79.3)	1.41 (1.14–1.76)	p < 0.001	1.22 (0.99–1.50)	**0.050**
**Average monthly income (UGX)**							
0-250000	477	302 (66.5)	175 (60.3)	1		1	
250,001–500,000	204	117 (25.8)	87 (30.0)	0.90 (0.79–1.03)	0.150	0.86 (0.75–0.98)	**0.029**
Above 500,000	63	35 (7.7)	28 (9.7)	0.87 (0.69–1.10)	0.252	0.83 (0.66–1.03)	0.098
**Duration of stay in the informal settlement**							
0–5 years	264	151 (33.3)	113 (39.0)	1		1	
6–10 years	145	86 (18.9)	59 (20.3)	1.03 (0.87–1.22)	0.678	0.96 (0.82–1.13)	0.681
More than 10 years	335	217 (47.8)	118 (40.7)	1.13 (0.99–1.29)	0.060	1.10 (0.96–1.25)	0.141
**Used psychoactive substance in the last 30 days**							
***Alcohol***							
No	191	82 (18.1)	109 (37.6)	1		1	
Yes	553	372 (81.9)	181 (62.4)	1.56 (1.31–1.86)	p < 0.001	1.43 (1.21–1.69)	**p < 0.001**
***Marijuana***							
No	358	198 (43.6)	160 (55.2)	1		1	
Yes	386	256 (56.4)	130 (44.8)	1.19 (1.06–1.34)	0.002	1.16 (1.02–1.31)	**0.017**
***Khat***							
No	335	177 (39.0)	158 (54.5)	1		1	
Yes	409	277 (61.0)	132 (45.5)	1.28 (1.13–1.45)	p < 0.001	1.25 (1.10–1.42)	**0.001**
***Heroin***							
No	730	444 (97.8)	286 (98.6)	1			
Yes	14	10 (2.2)	4 (1.4)	1.17 (0.83–1.64)	0.371		

## Discussion

Understanding the interaction between psychoactive substance use and sexual behaviour is central in reducing the burden of sexually transmitted infections (STIs), including HIV. This study investigated the predictors of sex under the influence of psychoactive substances among young people in informal settlements in Kampala, Uganda. Sex under the influence of psychoactive substances was high, and more prevalent among females, individuals aged 20–24 years, those earning a monthly income less than UGX 250,000 (≈ USD 67.0), and young people living without biological parents or guardians. Sex under the influence of psychoactive substances was also higher among alcohol, khat and marijuana users.

This study reports a high prevalence of sex under the influence of psychoactive substances among young people in informal settlements. The high prevalence of sex under the influence of psychoactive substances is not surprising, given the known impact of such substances on sexual behaviours [12]. Psychoactive substances such as alcohol are known to increase sex-related expectancies such as sexual pleasure, arousal and performance, and to reduce inhibitions such as lack of confidence in approaching a sex partner and difficulties in refusing to engage in sexual intercourse [12, 37, 57]. The combined effects of psychoactive substances on behaviour (sexual behaviour in this particular study) are explained by the myopia and expectancy theories. The Alcohol myopia theory suggests that the use of psychoactive substances impairs judgement, cognition and decision making [58], while the expectancy theory asserts that users of these substances are motivated by the outcomes they expect, which also include among others sexual performance and pleasure. It is the combination of desired benefits and impaired decision-making processes that drive young psychoactive substance users into risky sexual behaviours, including sex under the influence of psychoactive substances. The relationship between psychoactive substance use and sex while intoxicated is widely documented [12, 59, 60].

Our study revealed that users of alcohol, marijuana and khat had a higher prevalence of sex under the influence of psychoactive substances compared to the non-users of these substances. The use of marijuana and alcohol is associated with decreased sexual inhibitions (e.g., lack of confidence) and increased expectancies such as sexual arousal and sensations [12]. Hence, it is likely that young alcohol and marijuana users engaged in sex under the influence of psychoactive substances due to a reduction in sexual inhibitions, and an anticipated increase in sexual expectancies such as performance and pleasure. The use of alcohol and marijuana can alter rational decision making and cognition. These substances make it difficult for the users to evaluate who they should have sexual intercourse with, thus making sexual intercourse while intoxicated inevitable. Similar findings have been reported by Shamloul and Bella [61]. Although khat has not yet been linked to impairment of rational decision making [62], it is a known stimulant associated with increased energy levels, alertness [62], and user perceived benefits (such as, improved self-esteem and sexual performance). This could explain why khat users in this study may have had a higher prevalence of sex while intoxicated compared to non-users.

Females had a higher prevalence of sex under the influence of psychoactive substances compared to males. A significant proportion of females in our sample engaged in sex work as their main source of income. Indeed, a considerable portion of females in our study had had their last sexual contact with a sex client. Psychoactive substances such as alcohol, marijuana and khat are often used by sex workers to cope with abuse, traumatic experiences and to create a virtual reality within their mind. The fact that a significant proportion of the females in the current study had engaged in sex work, and more than half had engaed in multiple sexual relationships [48] also brings the role of sexual expectancies and inhibitions into context. There is evidence that females, especially those engaged in sex work, use psychoactive substances to improve sexual pleasure or euphoria, and performance including prolonging sexual intercourse with sex clients, and to boost their courage or confidence to approach sexual partners and clients [63]. Besides, psychoactive substances also increase sexual urge, make it difficult for the users to refuse sexual advance, and increase the likelihood of engaging in sexual intercourse [37, 64]. Existing gender norms with regards to sexual activity often imply that women are less likely to express their sexual desires compared to men. Consequently, women are more likely to have sex under the influence of psychoactive substances because they expect the substances to decrease sexual inhibitions and increase their confidence while expressing sexual desires, which would otherwise be constrained by existing gender and cultural norms [65]. In addition, physiological research has also shown that women are more affected by psychoactive substances such as alcohol because of their lower rates of gastric metabolism, body weights, and body mass indices [66]. This may lead to a greater impairment of their sexual decision-making abilities [67], including the decision to have sex under the influence of psychoactive substances.

Young people aged 20-24 years had a higher prevalence of sex under the influence of drugs compared to those aged 18–19 years. As individuals grow, there is often reduced social control such as parental monitoring and restrictions that would otherwise limit them from engaging in risky behaviours. Young people aged 20–24 years have more autonomy which makes them engage in sexual risk taking. There is also evidence of the 20–24 years age bracket being characterized by legal independence, curiosity and freedom to make their own decisions pertaining to psychoactive substance use and sex-risk taking among others [67]. This elevates their (those aged 20–24 years) risk to sex under the influence of psychoactive substances compared to their counterparts aged 18–19 years. Our study found that young psychoactive substance users that lived alone were more likely to have sex under the influence of alcohol compared to those living with their biological parents. The absence of positive parental guidance has the potential to influence risky sexual behaviours, such as early sex debut and sex under the influence of psychoactive substances [68, 69]. Therefore, staying with biological parents or guardians may be protective against sexual intercourse while under the influence of psychoactive substances. Similar findings have also been reported previously by TRáEN and Kvalem [70] and Waktole [71].

We noted that the married respondents and divorced/separated respondents had a higher prevalence of sex under the influence of drugs compared to those who were single. In society, marital satisfaction is often associated with sexual performance and satisfaction [72]. Therefore, individuals’ expectations about heightened sexual arousal and performance ability from using psychoactive substances, and reduced sexual inhibitions (lack of confidence to initiate sexual intercourse with their partner) could explain why married respondents had sex under the influence of psychoactive substances in the last 30 days. Divorced/separated respondents on the other hand, could have resorted to using psychoactive substances as a coping mechanism for the stress, which may have affected their perception, judgement and decision-making capacity, thereby leading them to having sexual intercourse while intoxicated. Our ideas on the relationship between marital status and sex under the influence of psychoactive substances are not concrete, and thus the need for further research.

Young people who earned an average monthly income below UGX 250,000 had a higher prevalence of sex under the influence of psychoactive substances compared to those who earned an average monthly income of UGX 250,000-500,000. Low income levels are known to increase vulnerability to undesirable sexual behaviours, including sex under the influence of psychoactive substances. Lower income levels make it difficult for young people to make independent decisions on matters related to their sexuality [73, 74]. Additionally, the low income levels in informal settlements also drive young people especially girls into sex work [75], which exposes them to psychoactive substances and their related effects on sexual behaviour. Our findings on the relationship between income levels and risky sexual behaviour (sex under the influence of psychoactive substances) has also been reported by other scholars [76, 77].

## Strengths and limitations

To the best of our knowledge, this is one of the few studies that has so far examined the prevalence of sex under the influence of psychoactive substances in informal settings in low-and middle-income countries. The study was conducted in the largest informal settlements in Kampala, a city that is representative of many in the global south [54], which makes the conclusions generalizable. The study provides evidence of the prevalence of sex under the influence of psychoactive substances among young psychoactive substance users across the different demographic strata such as sex, age category and level of education, and history of use of a wide range of psychoactive substances, which is often overlooked in other studies. Our study however, also had limitations that prospective studies need to address. Due to the cross-sectional nature of our study design, we did not estimate the independent effects of each of the psychoactive substances on sexual behaviour, yet, these substances are often complementary in nature i.e. the use of one psychoactive substance is often associated with using another. Psychoactive substances have diverse effects and taking a second substance can amplify or build on the effect of the first, or it may diminish or counteract it, or the combination of the two substances may be experienced as a different effect (WHO, 2018). Studies on sexual behaviour are sensitive and often subject to social desirability bias [51, 78, 79], and ours was no exception. Our intention was to use the national identification cards to verify the ages of the respondents, however, majority of the respondents lacked them. Consequently, we relied on self-reports which are also prone to bias. We made efforts to reduce social desirability bias by ensuring that interviews were conducted in settings that provided adequate privacy. Respondents were also reassured of confidentiality and anonymity during the course of the interviews.

## Conclusions

This study revealed a high prevalence of sex under the influence of psychoactive substances among young psychoactive substance users in informal settlements in Kampala. Across the socio-demographic strata, the prevalence was statistically higher among females, young psychoactive substace users aged 20–24 years, individuals who had attained the primary level of education and below, divorced and separated respondents, and those who were not living with their biological parents or guardians. The prevalence of sex under the influence of psychoactive substances was also statistically higher among individuals who had used alcohol, marijuana, oral tobacco and khat in the last 30 days. The predictors of sex under the influence of psychoactive substances were being female, being 20–24 years of age, being married or divorced/separated, not living with biological parents or guardians, and using alcohol, marijuana and khat in the last 30 days. Our study highlights the need for sexual and reproductive health programs to incorporate risk reduction interventions that aim to reduce sex under the influence of psychoactive substances among young people in informal settlements. There is also a need to sensitise young psychoactive substance users on the risk posed by sex while intoxicated.

## Data Availability

The datasets analysed during the current study are available from the corresponding author on reasonable request.

## List of abbreviations

**ABYM**: Adolescent Boys and Young Men
**HIV**: Human Immunodeficiency Virus
**RDS**: Respondent Driven Sampling
**RAs**: Research Assistants
**STIs**: Sexually Transmitted Infections
**UNAIDS**: The Joint United Nations Programme on HIV/AIDS
**UNODC**: United Nations Office on Drugs and Crime
